# Interfacial Polarization Phenomena in Compressed Nanowires of SbSI

**DOI:** 10.3390/ma15041543

**Published:** 2022-02-18

**Authors:** Anna Starczewska, Krystian Mistewicz, Mateusz Kozioł, Maciej Zubko, Danuta Stróż, Jan Dec

**Affiliations:** 1 Institute of Physics—Centre for Science and Education, Silesian University of Technology, 40-019 Katowice, Poland; krystian.mistewicz@polsl.pl; 2 Faculty of Materials Engineering, Silesian University of Technology, 40-019 Katowice, Poland; mateusz.koziol@polsl.pl; 3 Institute of Materials Science, University of Silesia, 41-500 Chorzów, Poland; maciej.zubko@us.edu.pl (M.Z.); danuta.stroz@us.edu.pl (D.S.); jan.dec@us.edu.pl (J.D.)

**Keywords:** impedance spectroscopy, interface, nanowire, ferroelectric, phase transition

## Abstract

The systematic studies of the extrinsic Maxwell–Wagner–Sillars polarization process in compressed antimony sulfoiodide (SbSI) nanowires are carried out by dielectric spectroscopy. The dielectric response is studied in temperature (100≤T≤350) K and frequency ($10^{-3}\leq f\leq 10^{6}$) Hz ranges. Dielectric functions commonly used for the analysis of dielectric spectra related to intrinsic polarization processes were applied in the elaboration of experimental data. It was found that the respective “semi-circles” in the Cole–Cole-type plots display a characteristic pear-like shape for the ferroelectric phase. On the other hand, the data for the paraelectric phase form symmetrical arcs. This response is effectively parametrized using the experimental Cole–Davidson and Cole–Cole functions fitted to the data obtained for the ferroelectric and paraelectric phases, respectively. It is deduced that the particular shape of spectra in the ferroelectric phase is due to spontaneous polarization, which is responsible for an asymmetric broadening of relaxation functions related to the interfacial polarization.

## 1. Introduction

It is common knowledge that “interfaces” are the basis of many electronic devices [1]. For example, in capacitors with a typical metal–insulator–metal structure, the metal–insulator interface prevents charge injection from metal into the insulator after the system reaches a steady state. On the other hand, the semiconductor–semiconductor *p*–*n* junction rectifies the carrier transport across the interface. Consequently, it results in the formation of an asymmetric charge distribution with respect to the interface. Another typical example of such an electronic device is represented by the metal–metal junction of two metals with different work functions. Here, in the vicinity of the metal–metal interface, a redistribution of charges leads to an electric double layer characterized by a so-called Volta potential difference.

The three representative examples mentioned above are sufficient to state that “interface” is a meeting area of two dissimilar materials where electric space charges are assembled. Consequently, a great deal of attention should be paid to such specific interfaces when fabricating modern electronic devices [2], in which the interfaces play a dominant role in their effective performance. In the particular case of two-material interfaces such as a metal–insulator interface, a semiconductor–semiconductor interface, or an insulator–semiconductor interface, a Maxwell–Wagner–Sillars (MWS) effect (also known as interfacial polarization mechanism) is considered as that which accounts for such a specific charge accumulation process [3,4,5,6]. The fundamentals of the MWS effect can be explained within the electromagnetic field theory by Maxwell’s equation $\nabla \cdot \vec{D}=\rho$, where $\vec{D}$ and *ρ* stand for densities of electric flux and charge, respectively. The details related to this interfacial polarization mechanism can be found in [6].

**Figure 1:**
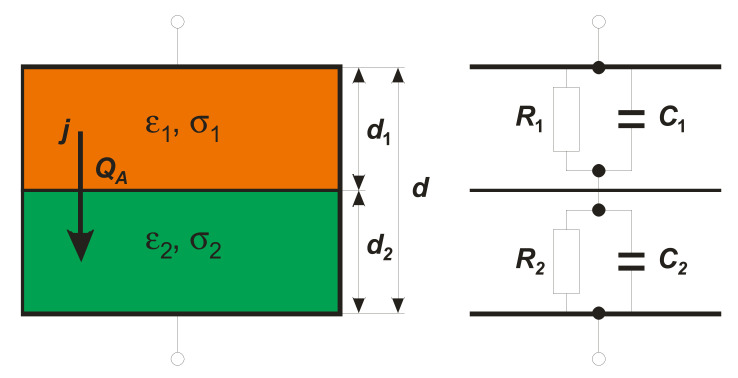
Scheme of two-layer condenser (**left**) and its equivalent circuit (**right**).

Let us consider the simplest case of an inhomogeneous structure in the form of a double-layer arrangement, as is presented in Figure 1. Here, each layer of nondispersive material is characterized by its permittivity $\varepsilon_{i}$, conductivity $\sigma_{i}$, and thickness *d_i_* (Figure 1). According to Maxwell’s electromagnetic theory, a total current flowing across such a structure is the sum of the conduction current and the Maxwell’s displacement current. Thus, there are two distinct paths for the current flow. This scenario can instructively be mimicked by a parallel equivalent *RC* circuit as seen in Figure 1, where *R_i_* represents a path due to conduction current and *C_i_* appears for a path due to displacement current. Here, *R_i_* and *C_i_* are given by $R_{i}=d_{i}/\left(AIf_{i}\right)$ and $C_{i}=\varepsilon_{0}\varepsilon_{i}A/d_{i}$, respectively ($\varepsilon_{0}$: permittivity of the free space, *A* cross-section). While the values of $R_{i}C_{i}$ define the individual time constants $\tau_{1}$ and $\tau_{2}$ of the equivalent circuits, respectively, the MWS relaxation time, $\tau_{\mathrm{MWS}}$, for the whole structure is given by [5,7]:

(1) $$\tau_{\mathrm{MWS}}=\frac{R_{1}R_{2}\left(C_{1}+C_{2}\right)}{R_{1}+R_{2}}=\varepsilon_{0}\frac{\varepsilon_{1}d_{2}+\varepsilon_{2}d_{1}}{\sigma_{1}d_{2}+\sigma_{2}d_{1}}.$$

It is seen that the MWS relaxation time depends not only on material parameters such as $\varepsilon_{i}$ and $\sigma_{i}$, but also on geometrical factors *d_i_*. Only in a very particular case of *d*_1_ = *d*_2_ does one arrive at the simplest expression [8]:

(2) $$\tau_{\mathrm{MWS}}=\varepsilon_{0}\frac{\varepsilon_{1}+\varepsilon_{2}}{\sigma_{1}+\sigma_{2}}$$

where $\tau_{\mathrm{MWS}}$ is defined only by material parameters. The above relaxation times characterize the process of charge accumulation at the two-material interface.

Indeed, starting from Gauss’ law, and using other known relations, one obtains [3]:

(3) $$\nabla \cdot \vec{D}=\nabla \cdot \varepsilon_{0}\varepsilon \vec{E}=\nabla \cdot \left(\frac{\varepsilon_{0}\varepsilon }{\sigma }\right)\vec{j}=\nabla \tau_{\mathrm{MWS}}\cdot \vec{j}=\rho$$

Here, *ε* and *σ* stand for effective permittivity and conductivity, respectively, of the two-layer condenser, which appears to the outside observer as a seemingly “single” dielectric medium possessing effective material parameters. As seen from Equation (3), the volume density *ρ* of the accumulated charge *Q_A_* is given as the inner product of the spatial gradient of relaxation time $\nabla \tau_{\mathrm{MWS}}$ and steady-state density of the current $\vec{j}$, which flows normally to the interface. Thus, continuing our considerations concerning the simple situation presented in Figure 1, the following relation is derived from Equation (3):

(4) $$\left(\tau_{2}-\tau_{1}\right)j=\sigma_{A}$$

where $\sigma_{A}$ is the surface density of charge on the interface. This relationship directly reveals the possibility for charge accumulation at the interface between two nondispersive materials, but distinctive only in time constants. When studying dielectric spectra, such charge accumulation appears in the form of a pure Debye-like dispersion step. The considered simple model, presented in Figure 1, predicts the existence of one unique relaxation time for the MWS polarization effect given by Equation (1). Real mesoscopic physical systems, e.g., compressed nanowires, consist of many differently oriented nano-sized grains different in material parameters and geometrical factors. This should lead to a distribution of relaxation times in such a complex system. The main goal of this paper is to carry out systematic studies of the extrinsic Maxwell–Wagner–Sillars polarization process in compressed antimony sulfoiodide (SbSI) nanowires. To this end, dielectric spectroscopy as a primary experimental technique is used. The dielectric response is studied in wide temperature (100≤T≤350 K) and frequency ($10^{-3}\leq f\leq 10^{6}$ Hz) ranges. The scientific novelty of the experimental data elaboration lies in applying available experimental dielectric functions commonly used to analyze dielectric spectra related to intrinsic polarization processes.

Another point is that, contrary to the commonly used procedure for the separate processing of real and imaginary permittivity data, we analyze our data in their natural complex space. It is expected that such a thorough elaboration of the interfacial polarization spectra will reveal a broad distribution of MWS relaxation times and, in addition, will deliver more reliable parameters characterizing the investigated interfacial polarization process. We are not aware of any paper in which the spectra of MWS polarization would be treated in such a way. The proper characterization of the distinctive MWS effect is crucial for developing new materials with exceptional properties.

Antimony sulfoiodide is a remarkable member of the ternary pnictogen chalcohalide family of materials [9]. SbSI is a photoferroelectric semiconductor with an anisotropic orthorhombic structure. It consists of double chains parallel to the [001] axis [10], which are held together by the van der Waals forces. The one-dimensional structure of the chalcohalide compound supports charge transport along the c-axis of the crystal and generates large internal electric fields [11]. The nanowires of SbSI exhibit a relatively low temperature of phase transition (*T_C_* = 291 K) and the indirect forbidden energy gap (*E_g_* = 1.86 eV) [12]. They also possess other interesting properties, such as piezoelectric [13], pyroelectric [14], photoelectrochemical [15], and photocatalytic [16,17]. Recently, SbSI has been recognized as a material suitable for application in piezoelectric nanogenerators for mechanical energy harvesting [18], efficient solar cells [19,20], electrochemical supercapacitors [21], and gas sensors [22,23]. The results of our investigations should shed some light on other possible applications of this unique material.

Until now, the one-dimensional nanostructures (nanowires, nanorods, nanoneedles) of SbSI have been fabricated using different methods, including the ball milling of single crystals [24], hydrothermal growth [25,26], vapor phase deposition [27], liquid exfoliation of the bulk crystals [28], solution processing [20], the sonication-heating route [29], and sonochemical synthesis [12,13,14,15,16,21,30]. The latter mentioned technology has many advantages. It is facile, cheap, and fast. The sonochemical preparation of SbSI nanowires can be completed within 2 h, whereas the ball milling of single crystals takes 50 h to obtain SbSI nanorods [24]. Moreover, sonochemical synthesis is performed at atmospheric pressure and relatively low temperature (323 K). It is in contrast to the hydrothermal method, which requires the application of an elevated pressure and a high temperature (453–463 K) [25]. The sonochemical method can be carried out in a single step. It is a great advantage in comparison to solution processing [20], which consists of two stages of material preparation. Finally, the tunability of the ultrasonic synthesis should be underlined. The morphology and properties of SbSI nanocrystals, obtained via this method, can be easily modified by using different solvents (e.g., ethanol [12], water [30], ethylene glycol [29]).

The results we publish here should stimulate engineers in their search for possible future applications.

## 2. Experimental Section

In this paper, SbSI nanowires were fabricated using a typical sonochemical procedure [22]. They were grown from antimony, sulfur, and iodine (Avantor Performance Materials, Gliwice, Poland) exposed to ultrasonic irradiation. The reagents were weighted in a stoichiometric ratio and immersed in ethanol, which was poured into a plastic vessel. The cylinder was inserted in a water bath of a VCX-750 ultrasonic reactor (Sonics & Materials, Inc., Newtown, CT, USA). Sonochemical preparation of SbSI gel was performed at a temperature of 323 K within 2 h. Detailed information on chemical reagents and used equipment can be found elsewhere [22,23].

The preparation of the compressed nanowire sample was like that reported previously [31,32,33]. It can briefly be described as follows. In the first step, the SbSI gel was dried for 10 h at an elevated temperature (313 K) to vaporize the ethanol. Thus, obtained raw SbSI xerogel was next placed into a steel cylinder, which served as a mold in the compression process. After filling with SbSI nanowires, the steel cylinder was closed with a piston. The mold was mounted into a 4469 Instron testing machine (Instron, Norwood, MA, USA). A sample in the form of a cylindrical pellet was prepared by compression of SbSI xerogel at room temperature by applying 160 MPa pressure and 5 mm/min loading bar speed.

The transmission electron microscopy (TEM) studies of SbSI nanowires were carried out using a JEOL high-resolution TEM (HRTEM) JEM 3010 microscope (JEOL USA Inc., Peabody, MA, USA). A 300 kV accelerating voltage was applied in these experiments. A scanning electron microscopy (SEM) and energy-dispersive X-ray spectroscopy (EDS) were used for the examination of the morphology and chemical composition of SbSI samples. It was done with the SEM microscope Phenom PRO X (Thermo Fisher Scientific, Waltham, MA, USA) equipped with an EDS detector.

For dielectric measurements, the major faces of the thus obtained pellet were covered with electrodes. The opposite sides of the sample were coated with a high-purity silver paint (SPI Supplies, West Chester, PA, USA). Next, the thin copper wires were attached to the sample electrodes. The prepared sample was then fastened into a stiff sample holder, which was next mounted in a high-efficiency research Janis cryostat STVP-200-XG (Janis Research Company, Woburn, MA, USA). This system uses static helium exchange gas to cool or warm the sample within the operating temperature range of 80–500 K. All the efforts are to avoid the influence of mechanical stresses on the investigated sample. As a result, the sample was mechanically free and had a solid electric contact. Before measurements, the sample was poled. After heating the sample to 350 K, a *dc* electric field of 12.6 V/mm was applied with subsequent cooling down to 100 K. The cooling rate was in the order of −1 K/min. At this temperature, the voltage was switched off and, after half an hour of waiting time, the capacitance measurements were initiated. The sample’s complex capacitance, ($\;C^{*}=C^{\prime }-iC^{\prime \prime }$), was measured with a Solartron 1260 impedance analyzer (Ametek Scientific Instruments, Leicester, UK) with a 1296 dielectric interface at frequencies $10^{-3}\leq f\leq 10^{6}$ Hz and temperatures 100≤T≤350 K. These temperatures mainly include a broadly accepted extended industrial grade for electronic devices comprised in the range of 230≤T≤360 K [34]. Above 350 K, SbSI behaves as a semiconductor; therefore, its reaction to the applied electric field can no longer be considered a dielectric response. This particularly concerns *dc* or low-frequency *ac* electric fields. On the other hand, the operating frequency of the electric field is also determined by the value of the MWS relaxation time. The amplitude of the *ac* probing voltage was in the order of 1 V. Temperature dependences of the capacitance were measured with a heating/cooling rate in the order of dT/dt=±0.25 K/min. The temperature in the cryostat was controlled to within ±0.01 K using a Lake Shore 335 temperature controller (Lake Shore Cryotronics, Inc., Westerville, OH, USA). The whole experimental protocol was managed with SMaRT Solartron software (Ametek Scientific Instruments, Leicester, UK).

## 3. Results and Discussion

**Figure 2:**
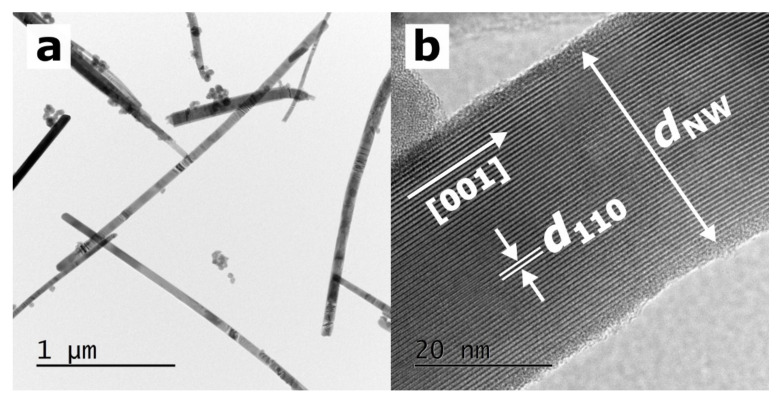
(**a**) TEM micrograph presenting a dozen SbSI nanowires and (**b**) HRTEM image of a selected SbSI nanowire. The white arrows indicate the [001] polar direction, nanowire thickness (*d*_NW_ = 33.6(16) nm), and interplanar spacing (*d*_110_ = 0.6517(20) nm), which corresponds to the interplanar spacing of (110) planes reported in the literature for SbSI [35].

Figure 2a shows the TEM image of SbSI nanowires, whose diameters range from approximately 20 to 200 nm and their lengths reach approximately a few micrometers. One nanowire was selected, and its morphology was analyzed (Figure 2b). The thickness of this nanowire, *d*_NW_ = 33.6(16) nm, and the interplanar spacing, *d*_110_ = 0.6517(20) nm, were determined. The value of *d*_110_ corresponds to the interplanar spacing of (110) planes reported in the literature for SbSI [35] within the experimental uncertainty. The SAED studies presented in [36] indicate that sonochemically synthesized SbSI nanowires usually grow along the polar [001] crystallographic c-direction.

Furthermore, it is evidenced that a thin amorphous shell covers the crystalline core of the SbSI nanowire. This unique attribute of SbSI nanocrystals has also been mentioned in other papers [37,38]. In the study [36], the specific surface area parameter was estimated at 75 m^2^/g, considering the average lateral dimensions and length of the sonochemically prepared SbSI nanocrystals.

**Figure 3:**
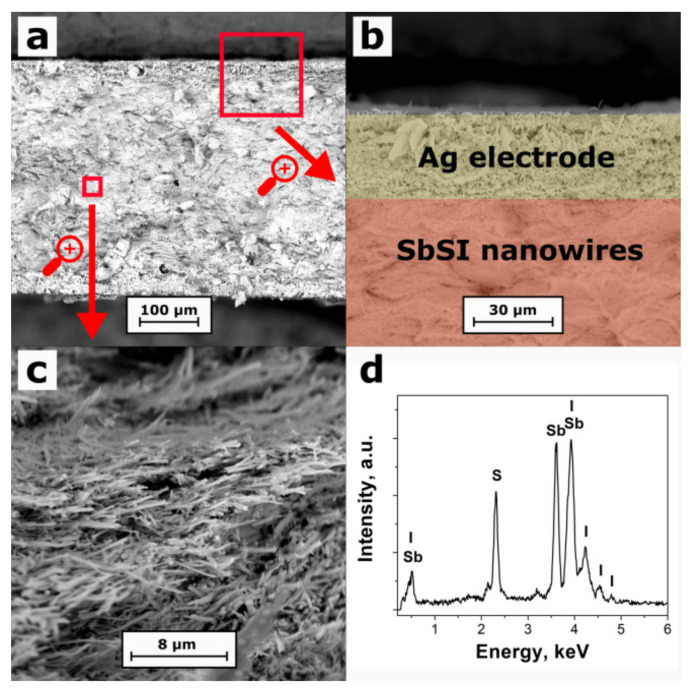
SEM images of the cross-section (**a**), edge (**b**) and interior (**c**) of the compressed SbSI nanowire sample, as well as its EDS spectrum (**d**).

A microstructure of the investigated SbSI sample is shown in Figure 3a–c. SEM investigations proved that the SbSI nanowires are randomly distributed in the examined sample. One can observe some voids (pores) in the pellet structure. Direct analysis proves that the SbSI nanowires contribute to only about 50% of the total sample volume, which is over 10 times higher than the packing factor of SbSI nanocrystals in the uncompressed xerogel volume [36]. The filling factor was evaluated considering the geometrical dimensions of the sample, its mass, and the density of the SbSI bulk crystal [39].

A typical EDS spectrum of the interior of the SbSI sample is presented in Figure 3d. It contains clear peaks, which were attributed to the chemical elements to calculate the chemical composition of the material. The atomic concentrations of 39.5%, 32.5%, and 28.0% (with an expanded coverage factor *k* = 3, the uncertainty of the order of 1.0%) were determined for antimony, sulfur, and iodine, respectively. No other chemical elements were found within the detection limit less than 0.1% of our EDS instrument. Thus, the evaluated real chemical composition of the sample is close to the stoichiometric one in which the percentage amount of each element should be the same (33.3%). A slight excess amount of antimony and deficiency of iodine in the examined material may be related to the presence of an amorphous shell on the SbSI nanowire surface (Figure 2b). Its chemical composition can slightly be different from a nanowire crystalline core [37,38].

**Figure 4:**
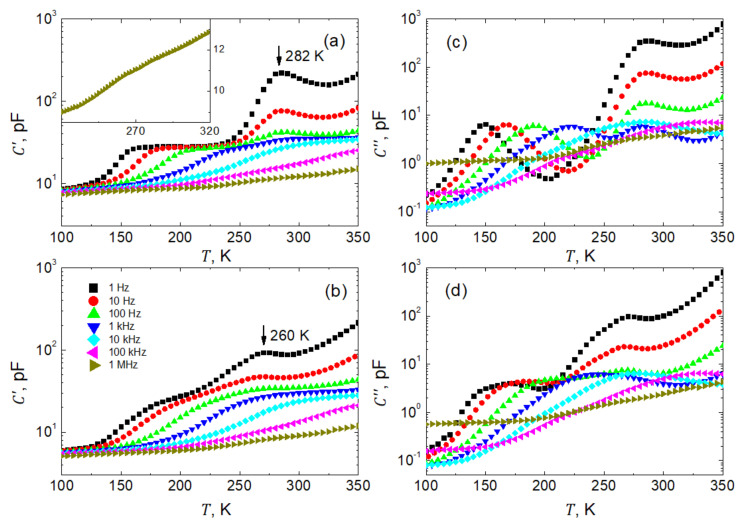
Temperature dependences of real, *C*′ (**a**,**b**), and imaginary, *C*″ (**c**,**d**), parts of the complex capacitance of SbSI sample measured at decade stepped frequencies within $10^{0}\leq f\leq 10^{6}$ Hz during heating (**a**,**c**) and cooling (**b**,**d**) run, respectively. For the sake of clarity, only every fifth experimental point is shown. The inset shows the *C*′(*T*) dependence for 1 MHz.

In order to optimize the systematic investigations of dielectric spectra, temperature dependences of capacitance are measured for initial recognition of possible peculiarities in dielectric response. Figure 4 presents such temperature dependences of the real (Figure 4a,b) and imaginary (Figure 4c,d) parts of the complex capacitance $C^{*}=C^{\prime }-iC^{\prime \prime }$. The data were obtained by heating (Figure 4a,c) and cooling (Figure 4b,d), for frequencies $10^{0}\leq f\leq 10^{6}$ Hz in decadic order, and temperatures 100≤T≤350 K. Here, due to the complexity in the microstructure of the investigated sample, we decided not to rescale the capacitance data into permittivity. The point is that, due to the relatively high porosity and a considerable number of interfaces in the sample, the permittivity data would be a very inappropriate material parameter of the compressed nanowire SbSI sample.

The capacitance data on the heating run had been acquired after the prior polling procedure of the SbSI sample as described earlier. The $C^{\prime }\left(T\right)$ curves reveal the existence of two distinct anomalies visible as shoulders and smeared maxima (Figure 4a,b). Here, one should mention first that the anomalies in capacitance as observed on heating, i.e., in the first run after poling, manifest themselves more plainly than in the subsequent measurements on cooling.

As seen in Figure 4a, the shoulder-like anomalies gradually smear, shift towards higher temperatures, and finally leave our frequency window when increasing the frequency of the probing voltage (Figure 4a,b). A possible mechanism of this contribution to the dielectric response will be discussed later.

On the other hand, maxima on the $C^{\prime }\left(T\right)$ curves occur at nearly fixed 282.0±1.5 K temperatures on heating and 260.0±1.5 K on cooling (Figure 4a,b), respectively. Such a temperature fixation of the maxima manifests the phase transition experienced by the individual nanowires. The bulk SbSI is known to undergo a ferroelectric first-order phase transition at $T_{C}=295$ K [40] with a thermal hysteresis as an inherent feature of this kind of transformation. This thermal hysteresis between heating/cooling runs is also present in our sample. As observed in our sample, the lower phase transition temperatures may result from slight non-stoichiometry of individual nanowires and internal strains generated by thermal expansion and/or the structural phase transformation (at the first-order phase transition, a jump-like change in volume of the elementary unit cell is expected). It is well established in the literature (see, e.g., [41]) that external pressure shifts the temperatures of the first-order phase transition towards lower $T_{C}$, widens the respective permittivity peaks and reduces the maximum permittivity value in comparison to the mechanically free sample. This is precisely what we observe in our case of a compressed nanowire sample.

Moreover, single-crystalline SbSI is also characterized by a high value of anisotropy in permittivity. The ratio of permittivity measured along the polar ferroelectric axis to that measured in the perpendicular direction is as large as 2000 [40]. Thus, having such an inhomogeneous system of disordered nanocrystals, one measures an effective response that significantly differs from that known for well-defined single crystalline samples. It is also worth mentioning that, depending on the quality of the single-crystalline samples, $T_{C}$ may vary between 283 and 298 K [42].

Significant dispersion of the permittivity in the vicinity of the phase transition point (Figure 4a,b) is related to the contribution of ferroelectric domains and domain walls to the dielectric response. The permittivity decreases by more than one order of magnitude when increasing the frequency of the probing field. At the same time, the distinctness of the peaks decreases, which also interferes with the moving shoulder-like anomaly, in addition. These two anomalies in $C^{\prime }\left(T\right)$ curves overlap at higher frequencies, and the resulting peculiarity becomes hardly seen. See the inset in Figure 4a, which presents a respective fragment of the $C^{\prime }\left(T\right)$ measured at a frequency of 1 MHz.

All the above remarks regarding the temperature dependences of the real part of the capacitance (Figure 4a,b) also apply to the imaginary $C^{\prime \prime }\left(T\right)$ part of the capacitance (Figure 4c,d), respectively. As typically for losses, both anomalies are displayed as bell-shaped curves. Unluckily, these textbook profiles appear partially spoiled at higher temperatures by the enhanced electrical conductivity of the investigated sample.

**Figure 5:**
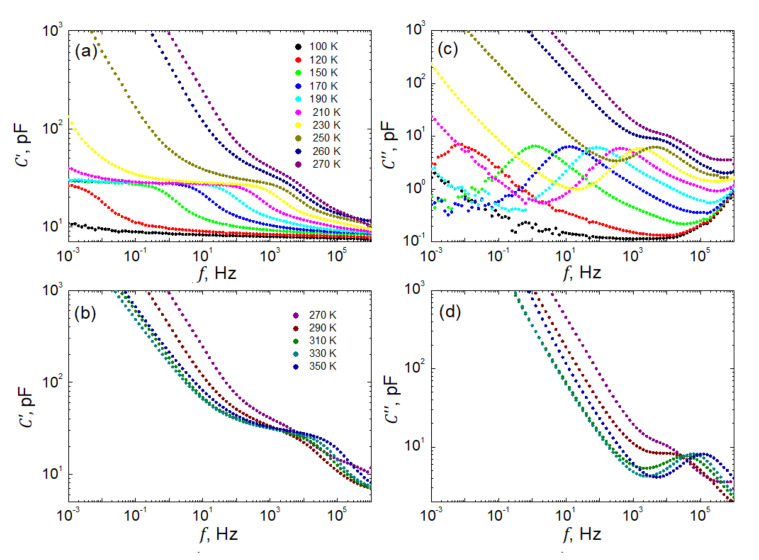
Representatively selected frequency dependences of real, *C*′, (**a**,**b**) and imaginary, *C*″, (**c**,**d**) parts of the complex capacitance of SbSI sample at temperatures T≤270 K (**a**,**c**) and T≥270 K (**b**,**d**).

Figure 5 presents representatively selected spectra of real $C^{\prime }\left(f\right)$, and imaginary, $C^{\prime \prime }\left(f\right)$ parts of the complex capacitance measured every 5 K within the temperature range of 100≤T≤350 K. As seen in Figure 5a, below 100 K the investigated sample is almost nondispersive in our frequency window $10^{-3}\leq f\leq 10^{6}$ Hz. Only a slightly monotonic increase in capacitance when decreasing the frequency of the probing field is observed. Around the temperature of 100 K, a well pronounced dielectric step emerges, which then shifts towards higher frequencies when increasing the temperature. Remarkably, the so-called static capacitance, $C_{s}^{\prime }\sim 30$ pF, as measured at frequencies $10^{-3}\leq f\leq 10$ Hz, practically does not depend on temperature. This very typical behavior is perturbed within the temperature span around the phase transition, and next, another dielectric step is observed (see Figure 5b). Such a disturbance is due to some instability in the system undergoing structural and ferroelectric phase transformation. Another deviation from the regularity, as observed in the low-*f* parts of the spectra, is due to *dc* electric conduction. It is evident as a steep increase in capacitance when decreasing the frequency of the probing electric field. This apparent contribution to the dielectric relaxation significantly enhanced by shifting towards higher frequencies when the temperature increases.

Spectra of the losses, as represented in Figure 5c,d by the imaginary part of the capacitance $C^{\prime \prime }\left(f\right)$, can be discussed in an analogical way. However, in this case, one observes typical bell-shaped curves gradually shifting towards higher frequencies when temperature increases.

**Figure 6:**
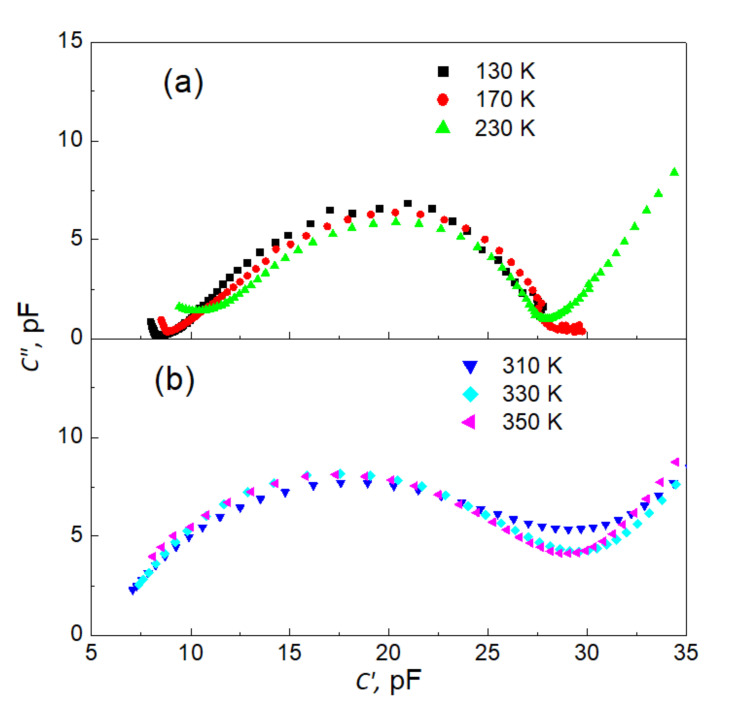
Representatively selected dispersion curves on the complex plane *C*″ (*C*′) for ferroelectric (**a**) and paraelectric (**b**) phase.

Additional information on the character of the investigated relaxation can be extracted from dispersion curves plotted on the complex plane (Figure 6a,b). In Figure 6, only representative curves for ferroelectric (Figure 6a) and paraelectric (Figure 6b) phases are shown.

These two families of curves qualitatively differ in their shape. While the “semi-circles” for the ferroelectric phase display a characteristic pear-like shape, the data for the paraelectric phase form symmetrical arcs. These two features signify deviations from the model Debye-like relaxation function [8,43]. To parametrize the observed MWS relaxation process, an appropriate fitting procedure was carried out—the experimental Cole–Davidson function [8,44]:

(5) $$C^{*}\left(\omega \right)=C_{\infty }+\frac{C_{S}-C_{\infty }}{{\left(1+i\omega \tau_{CD}\right)}^{\beta }}=C_{\infty }+\frac{\Delta C}{{\left(1+i\omega \tau_{CD}\right)}^{\beta }}$$

where $\Delta C=C_{S}-C_{\infty }$ stands for relaxation strength with $C_{S}=\underset{\omega \tau \ll 1}{\lim}C^{\prime }\left(\omega \right)$ and $C_{\infty }=\underset{\omega \tau \gg 1}{\lim}C^{\prime }\left(\omega \right)$ and was fitted to the data represented by the pear-like course. The shape parameter *β*$\left(0<\beta \leq 1\right)$ describes an asymmetric broadening of the relaxation function for angular frequencies $\omega >1/\tau_{CD}$, where $\tau_{CD}$ is the Cole–Davidson relaxation time. For β=1, the Debye relaxation function is obtained. It should be mentioned here that the characteristic relaxation time $\tau_{CD}$ does not coincide with the relaxation time extracted from the position of maximal loss.

On the other hand, the Cole–Cole function [8,44]

(6) $$C^{*}\left(\omega \right)=C_{\infty }+\frac{C_{S}-C_{\infty }}{1+{\left(i\omega \tau_{CC}\right)}^{\alpha }}=C_{\infty }+\frac{\Delta C}{1+{\left(i\omega \tau_{CC}\right)}^{\alpha }}$$

where 0<α≤1 leads to the symmetrical broadening of the relaxation function, was fitted to the data compatible with the symmetrical arcs. Here, the Cole–Cole relaxation time $\tau_{CC}$ gives the position of maximal imaginary capacitance *C*″ as $\omega_{p}=2\pi f_{p}=1/\tau_{CC}$. For α=1, the Debye function is recovered again.

**Figure 7:**
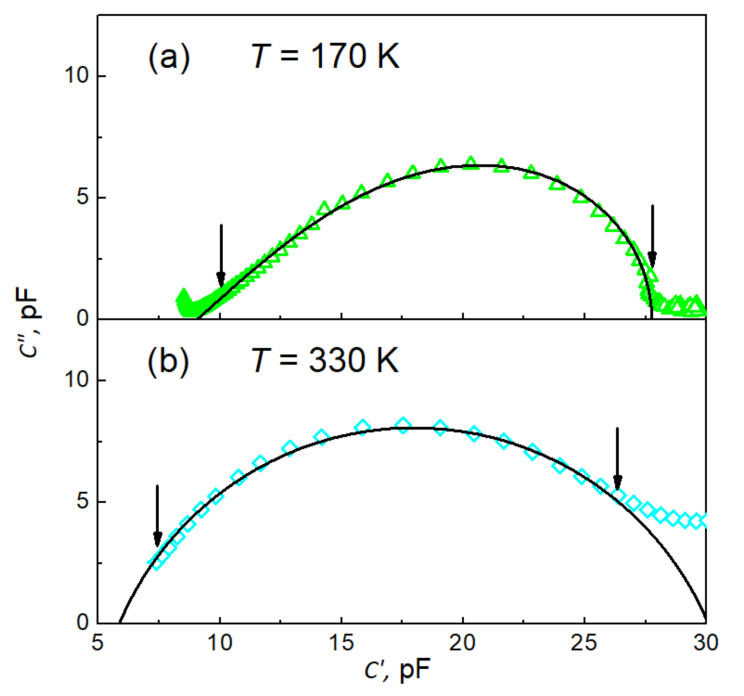
Cole–Cole-type plots $C^{\prime \prime }$ vs. $C^{\prime }$ of experimental data (open symbols) and their best fits (black solid lines) (**a**) to Equation (5) with *C*_∞_ = 90.52(89) pF, Δ*C* = 18.69(10) pF, *τ*_CD_ = 25.84(56) ms, and *β* = 0.4674(74) and (**b**) to Equation (6) with *C*_∞_ = 5.86(28) pF, Δ*C* = 24.22(43) pF, *τ*_CC_ = 2.83(40) ms, and ¦*α* = 0.7449(26). The vertical arrows indicate the range of data used for the fits.

All the fits were carried out on the complex plane; thus, the real and imaginary capacitance data were exploited simultaneously. This approach ensures higher reliability of the fitting procedure rather than fitting real and imaginary parts of capacitance separately. The peculiarities of the fits are illustrated in Figure 7a,b, where typical examples for both types of dependences are demonstrated. The solid line in Figure 7a represents the best fit of Equation (5). The fitting parameters $C_{\infty }=9.52\left(89\right)$ pF, $\Delta C=18.69\left(10\right)$ pF, $\tau_{CD}=25.84\left(56\right)$ ms, and $\beta =0.4674\left(74\right)$ are for the data measured at 170 K. The fitting was carried out within the data range marked by vertical arrows, and next the line was extrapolated outside the fitting range to draw an entire dependence. As seen in Figure 7a, the quality of the fit is highly satisfactory within experimental uncertainties. Similarly, the solid line in Figure 7b represents the best fit of Equation (6) with fitting parameters $C_{\infty }=5.86\left(28\right)$ pF, $\Delta C=24.22\left(43\right)$ pF, $\tau_{CC}=2.83\left(40\right)$ ms and $\alpha =0.7449\left(26\right)$ for the data measured at 330 K. An almost perfect fit is reached again.

**Figure 8:**
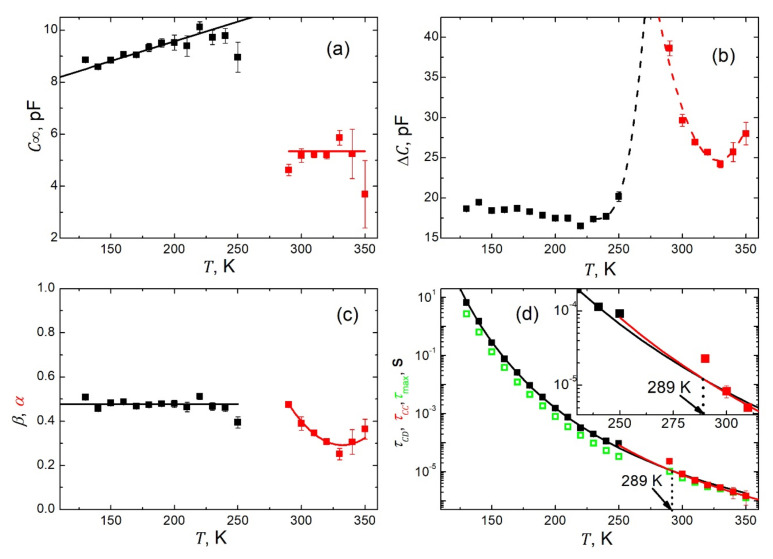
Temperature dependences of the fitting parameters from the best fits of the $C^{\prime \prime }$ vs. $C^{\prime }$ data to Equations (5) and (6), respectively. (**a**) High frequency limit of the capacitance $C_{\infty }=\underset{\omega \tau \gg 1}{\lim}C^{\prime }\left(\omega \right)$. The solid lines are to guide the eyes. (**b**) Relaxation strength $\Delta C=C_{S}-C_{\infty }$. The dashed lines anticipate possible dependences around the phase transition point. (**c**) Shape parameters *β* (black squares) and α (red squares). The solid lines are to guide the eyes. (**d**) Cole–Davidson $\tau_{CD}$ and Cole–Cole $\tau_{CC}$ relaxation times (black and red squares respectively). The solid lines represent the best fit to Arrhenius law $\tau =\tau_{0}exp\left(E_{a}/k_{B}T\right)$ with $\tau_{0}=2.31\left(28\right)\cdot 10^{-10}$ s and $E_{a}=0.2708\left(18\right)$ eV for the black curve, and $\tau_{0}=0.30\left(13\right)\cdot 10^{-10}$ s and $E_{a}=0.322\left(12\right)$ eV for the red one. For comparison, the relaxation time $\tau_{\max}$, determined from the maximum of the imaginary part of the capacitance (green open squares), is shown.

In total, we carried out 13 fits for spectra taken in the ferroelectric phase within the temperature range of 100≤T≤250 K and seven fits for the data acquired in the paraelectric phase within 290≤T≤350 K. The resulting temperature dependences of the respective fitting parameters are presented in Figure 8. Figure 8a presents the temperature dependence of the high frequency limit of the capacitance $C_{\infty }$. While this quantity displays a weak, almost linear rise with increasing temperature in the ferroelectric phase, it is almost constant in the paraelectric state. However, in the vicinity of the phase transition, a drop in capacitance takes place by about 50%. This could be ascribed to the vanishing of spontaneous polarization and thus one crucial contribution to the dielectric response vanishes. The relaxation strength ΔC manifests typically for low frequency capacitance (see Figure 4a) and temperature dependence with an anticipated maximum in the vicinity of the phase transition point (Figure 8b). The shape parameter, *β*, as extracted for the polar state, is practically (within uncertainties) temperature independent on the level of 0.48 (Figure 8c). Afterward, in the paraelectric phase where the Cole–Cole-type plots become symmetric, the respective shape parameter *α* reveals quite a significant temperature dependence (Figure 8c). At this point, it is worth noting that the pear-like shape of the Cole–Cole-type plots is due to the presence of the spontaneous polarization pertaining to the ferroelectric state. The relaxation times formally follow the Arrhenius law $\tau =\tau_{0}exp\left(E_{a}/k_{B}T\right)$, as represented by the black fitted line with the pre-exponential factor $\tau_{0}=2.31\left(28\right)\cdot 10^{-10}$ s, and activation energy $E_{a}=0.2708\left(18\right)$ eV for the ferroelectric state, and the red one with $\tau_{0}=0.30\left(13\right)\cdot 10^{-10}$ s and $E_{a}=0.322\left(12\right)$ eV for the paraelectric phase, respectively (Figure 8d). The extrapolated fitting curves intersect at a temperature T=289 K close to the phase transition point.

**Figure 9:**
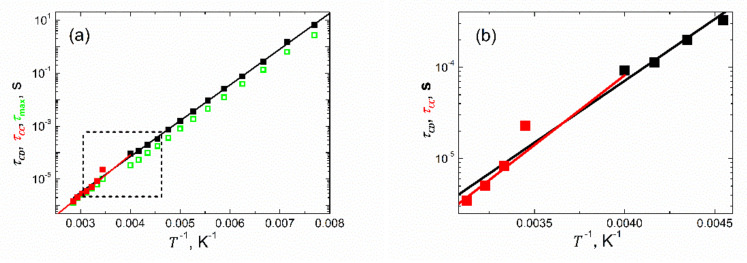
Arrhenius-type presentation of the relaxation times shown in Figure 8d. (**b**) Data replotted from the area marked with a rectangle in (**a**) to visualize the intersection of the fitting lines.

Many authors prefer to determine the relaxation times from the maximum of the imaginary part of the permittivity/capacitance, as observed on the respective spectra. Here, we decided to carry out such an exercise. The values of relaxation times thus obtained are shown in Figure 8d as green open symbols. It is seen from Figure 8d that while in the paraelectric state (where symmetric Cole–Cole-type plots are observed, see Figure 7b), the two sets of relaxation times overlap. In the ferroelectric phase (with asymmetric Cole–Cole-type plots, see Figure 7a), the relaxation times determined from the maximum of the imaginary part of the capacitance are systematically lower than those obtained in the fitting procedure. Fundamentals for such behavior can be found in [8,43]. In addition, these two sets of relaxation times $\tau_{CD}$ and $\tau_{CC}$ display slight but essential differences in their temperature dependences, which are expressed in another fashion in Figure 9, presenting relaxation times as a function of an inverse of temperature. These differences are revealed thanks to the appropriate selection of the parametrization functions used for the disparate Cole–Cole-type plots. Thus, the advantage of our data processing procedure is demonstrated.

Furthermore, it should be emphasized at this point that the Cole–Davidson $\tau_{CD}$ and Cole–Cole $\tau_{CC}$ relaxation times have a different meaning within their distinctive experimental models [8,43]. While $\tau_{CD}$ represent here the longest relaxation times [8] in the set of MWS relaxation times (see Introduction) within 100≤T≤250 K, $\tau_{CC}$ extracted within 290≤T≤350 K are the average [44] MWS relaxation times of the system. This gives reasons for the disclosed difference in the values of the fitting parameters related to the temperature dependences of $\tau_{CD}$ and $\tau_{CC}$, respectively.

A gap in the fitting parameters, as seen in Figure 8 and Figure 9, in the temperature range of 250<T<290 K is due to the overlapping of two relaxation models distinctive for two different states of the sample. This resulted in an unstable fit with highly unreliable values of fitting parameters.

## 4. Conclusions

In summary, the compressed nanowire SbSI system features significant Maxwell–Wagner–Sillars interfacial polarization phenomena, which mask to some degree the proper dielectric response of the investigated sample. The observed interfacial MWS polarization process can effectively be parametrized using the experimental Cole–Davidson and Cole–Cole functions fitted to the data collected in the ferroelectric and paraelectric phases, respectively. While the presence of the spontaneous polarization seems to be responsible for an asymmetric broadening of relaxation functions related to MWS polarization in the ferroelectric state, the paraelectric state without spontaneous polarization is characterized by the symmetric broadening of relaxation functions. The effect of the spontaneous polarization on the MWS polarization process in compressed nanowires of SbSI is also clearly reflected in the temperature dependences of the fitting parameters (see Figure 8). This is because of the high reliability of the fitting parameters extracted during the respective data treatment carried out in the complex space. We are not aware of any paper presenting results of investigations of the MWS polarization in SbSI. Our new methodology for data treatment presented in this paper may be used for description of the MWS polarization process in any other materials.

## Data Availability

The data presented in this study are available upon request from the corresponding author.
